# Alzheimer’s Disease Prediction Using Fisher Mantis Optimization and Hybrid Deep Learning Models

**DOI:** 10.3390/diagnostics15121449

**Published:** 2025-06-06

**Authors:** Sameer Abbas, Mustafa Yeniad, Javad Rahebi

**Affiliations:** 1 Computer Engineering Department, Ankara Yildirim Beyazit University, 06010 Ankara, Türkiye; samir88eng@gmail.com (S.A.); myeniad@gmail.com (M.Y.); 2 Software Engineering Department, Istanbul Topkapi University, 34662 Istanbul, Türkiye

**Keywords:** Alzheimer’s disease diagnosis, CNN, feature selection, Fisher Mantis Optimization algorithm

## Abstract

**Background/Objectives**: Alzheimer’s disease (AD) is a progressive neurodegenerative disorder causing memory, cognitive, and behavioral decline. Early and accurate diagnosis is critical for timely treatment and management. This study proposes a novel hybrid deep learning framework, GLCM + VGG16 + FMO + CNN-LSTM, to improve AD diagnosis using MRI data. **Methods**: MRI images were preprocessed through normalization and noise reduction. Feature extraction combined texture features from the Gray-Level Co-occurrence Matrix (GLCM) and spatial features extracted from a pretrained VGG-16 network. Fisher Mantis Optimization (FMO) was employed for optimal feature selection. The selected features were classified using a CNN-LSTM model, capturing both spatial and temporal patterns. The MLP-LSTM model was included only for benchmarking purposes. The framework was evaluated on The ADNI and MIRIAD datasets. **Results**: The proposed method achieved 98.63% accuracy, 98.69% sensitivity, 98.66% precision, and 98.67% F1-score, outperforming CNN + SVM and 3D-CNN + BiLSTM by 2.4–3.5%. Comparative analysis confirmed FMO’s superiority over other metaheuristics, such as PSO, ACO, GWO, and BFO. Sensitivity analysis demonstrated robustness to hyperparameter changes. **Conclusions**: The results confirm the efficacy and stability of the GLCM + VGG16 + FMO + CNN-LSTM model for accurate and early AD diagnosis, supporting its potential clinical application.

## 1. Introduction

Alzheimer’s disease (AD) is the most common cause of dementia in the world, affecting over 55 million people globally—a figure expected to triple to 152 million by 2050 [1,2,3,4,5]. This irreversible neurodegenerative disorder progressively deteriorates memory, cognition, and behavior, eventually leading to complete dependency and death [5,6,7,8,9]. The global socioeconomic burden of AD is enormous, with annual costs of over $1 trillion, underscoring the urgent need for timely and accurate diagnostic methods [1,2,5,10]. Current diagnostic techniques include clinical assessment, neuropsychological examination, and neuroimaging techniques such as MRI and CT scans [11,12,13,14,15,16,17,18].

**Figure 1:**
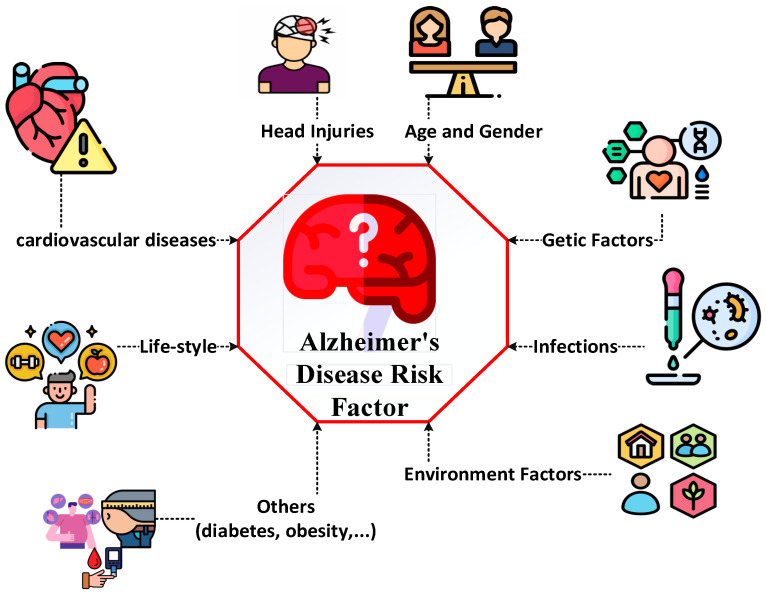
Elements influencing the development of AD.

While informative, these methods are often invasive, costly, and insufficiently sensitive for early-stage detection [5,11,14,15]. Biomarkers such as hippocampal atrophy, temporal lobe degeneration, and abnormal EEG frequency rhythms have shown clinical relevance, yet few models utilize them in a fully integration manner [17,19,20,21,22,23,24]. Furthermore, hippocampal and temporal lobe atrophy are MRI-derived, while rhythmic EEG features are extracted non-invasively—highlighting the need for a diagnostic model that merges both modalities [17,22,23,24,25,26,27]. There are various factors in the occurrence of AD, which are shown in Figure 1.

In recent years, machine learning (ML) techniques have been applied to AD diagnosis, especially using EEG signals due to their non-invasiveness and cost effectiveness [28]. However, most ML-based approaches struggle with generalization, overfitting, and limited interpretability. Furthermore, the classification of EEG signals for AD diagnosis, as reported in the recent literature [29,30], remains underexplored in hybrid models combining EEG with structural imaging data.

This study proposes a novel hybrid diagnostic framework that integrates frequency-specific EEG features and CT imaging data. A modified Fisher Mantis Optimization algorithm is employed to perform feature selection. This algorithm is tailored for efficiently selecting features in complex biomedical datasets with high dimensionality and variability. Although FMO has been employed in generic optimization tasks, its tailored adaptation for EEG-CT feature integration in early AD detection is novel.

The proposed method is classified by a neural network model that balances performance with interpretability. The contributions of this study are as follows:

- Emphasizing the diagnostic relevance of EEG frequency bands, particularly those associated with early memory impairment—an area seldom found in prior AD models.
- Using an improved FMO algorithm for robust feature selection across multimodal data, improving model generalizability and efficiency.
- Developing an interpretable AI-based pipeline that combines non-invasive neurofeedback data with imaging-based biomarkers to enable accurate and practical AD classification.

In summary, this study targets limitations of existing diagnostic methods, combining low-cost non-invasive techniques with modern AI strategies, contributing a practical and scalable framework for early Alzheimer’s disease detection.

### Literature Review

The literature review on Alzheimer’s disease (AD) diagnosis reveals a recent trend towards the utilization of machine learning (ML) and feature selection (FS) techniques to improve classification performance and reduce data dimensions. Given the high-dimensional nature of neuroimaging and clinical data, FS has become an essential step in developing reliable diagnostic models. This section only reports studies that combine FS with classification approaches. In [31], a hybrid method was introduced using Principal Component Analysis (PCA) for feature reduction and Fisher’s Linear Discriminant for classification. The approach achieved high performance, with 96.32% accuracy, 94.11% sensitivity, and a feature reduction rate of 98.52%. Reference [32] employed a Genetic Algorithm (GA) for feature selection and a Support Vector Machine (SVM) for classification, achieving 93.01% accuracy and a 96.80% feature reduction rate.

These studies demonstrate that the pipeline presented in [33] involved manual feature extraction from MRI scans, followed by classification using a Support Vector Machine (SVM), achieving an accuracy of 93.2% and a feature retention rate of 93.3%. These results underscore the potential of traditional approaches, although they may be limited in scalability and automation compared to the deep learning-based models proposed in our study. Pixel and voxel-based FS methods were explored in [34], where a classifier-independent method produced 98% accuracy and 95% FS efficiency, indicating that conventional ML classifiers could be bypassed by direct spatial domain analysis. In [35], Random Forest (RF) was employed not only for classification but also as an embedded FS tool, enhancing interpretability while maintaining robust classification capability. Beyond imaging, [36] explored speech-based feature extraction, focusing on linguistic patterns to classify early-stage AD cases.

While achieving reasonable accuracy (~91%), such domain-specific features may lack generalizability across datasets. Two comprehensive and recent reviews provide essential insights into the current trends. First, the study [37] aims to identify reliable biomarkers and therapeutic targets for Alzheimer’s disease by developing a robust multi-filter gene selection framework that integrates biological and machine learning methods to improve diagnosis accuracy. The study proposes and validates an aggregative gene selection approach combining hub gene ranking with feature selection algorithms, prioritizing predictive genes on independent data. Second, this study applies TL to improve Alzheimer’s diagnosis accuracy using an evolving MRI database, boosting accuracy from 63% to 99% when historical scans are available and up to 83% by fine-tuning 2D models for 3D data [38].

Despite the advancements, several studies do not apply FS techniques, instead relying on feature extraction via convolutional networks without clear selection mechanisms. This can lead to feature redundancy and reduced generalizability. A novel binary variant of the Akhundak Algorithm for FS was proposed by Salehi et al. [39] which is designed to optimize discrimination by eliminating redundant and irrelevant features. This FS method is evaluated in conjunction with SVM and Artificial Neural Networks (ANNs), aiming to strike a balance between sensitivity, feature reduction, and accuracy. This framework not only simplifies the model but also improves classification efficiently in higher dimensional AD data. Table 1 shows a comprehensive comparison of the recent state-of-the-art methods (GA: a Genetic Algorithm, SVM: Support Vector Machine, PCA: Principal Component Analysis, MR: Magnetic Resonance, PET: Positron Emission Tomography, CSF: Cerebrospinal Fluid, ICA: Independent Component Analysis, FA: Fractional Anisotropy, TBSS: Tract-Based Spatial Statistics, RF: Random Forest, DISR: Double Input Symmetrical Relevance, TL: Transfer Learning, CNN: Convolutional Neural Network), for Alzheimer’s disease diagnosis, summarizing their feature selection methods, classifiers, datasets, performance metrics, and key contributions.

## 2. Materials and Methods

### 2.1. Overview of the Computational Framework

This section explains how the proposed approach was applied to MRI scans of Alzheimer’s patients using the MATLAB 2024a deep learning toolbox. The MRI scans were labeled as indiactive of Alzheimer’s or normal (control), and this section includes evaluation parameters and comparative analysis.

For this purpose, a comprehensive, automated, and modular computational framework is developed that includes the steps of MRI data loading, preprocessing, feature extraction, and feature selection, and finally classification using a hybrid CNN-LSTM model.

### 2.2. Dataset Description

**Figure 2:**
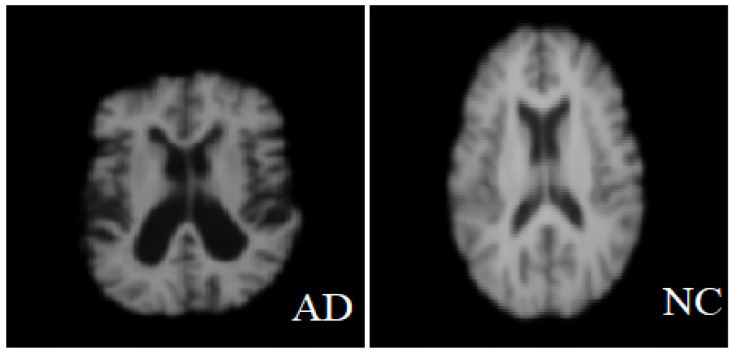
Two examples of malignant and benign Alzheimer’s images [42].

The MRI images used in this study were obtained from two publicly available datasets: the Alzheimer’s Disease Neuroimaging Initiative (ADNI) and minimal interval resonance imaging Alzheimer’s disease (MIRIAD). Official permission was secured for using the ADNI dataset, and no external image sources were used. The ADNI dataset is designed to monitor the early progression of Alzheimer’s disease and includes MRI scans with 128 sagittal slices, typically formatted as 256 × 256 matrices. It comprises data from 741 participants, including 314 AD patients and 427 normal controls subjects. The MIRIAD dataset includes MRI images of 46 AD patients and 23 normal controls, scanned at time points between 2 weeks and 2 years [40,41]. In this study, all image processing, model training, and evaluation were performed exclusively on sagittal slices due to their consistency and suitability for analysis. Axial slices were used solely for visualization purposes in Figure 2, given their higher display quality. The figure illustrates examples of benign and malignant images from both ADNI and MRTIAD datasets, helping to visually distinguish between the two diagnostic categories. Table 2 groups the samples by dataset and class (Alzheimer’s disease—AD and control—NC), showing the total number of samples for each category, and then shows their distribution into training (70%) and test (30%) sets.

The ADNI dataset was chosen based on its wide clinical acceptance and detailed longitudinal imaging for early diagnosis of Alzheimer’s disease. The MIRIAD dataset complements ADNI by offering multiple time-point scans to evaluate model stability through temporal changes.

### 2.3. Data Preprocessing and Feature Extraction

The MRI data underwent a structured preprocessing pipeline to enhance image quality and ensure consistency across samples. Initially, all images were spatially registered to a common reference template to correct for positional differences.

Noise reduction was then applied to suppress unwanted variations while preserving anatomical structures. Intensity normalization was performed to scale the pixel values to the standard range, facilitating uniform feature representation. To focus on relevant brain regions, a combination of thresholding and morphological operations was used for effective segmentation and removal of non-brain tissues.

Following preprocessing, a rich features set was extracted from each image to support robust classification. Three categories of features were considered:

1. Deep features: High-level representations were obtained by passing each MRI scan using a pretrained CNN. Deep features encode highly abstract and discriminative patterns.
2. Texture features: These are statistical measures that capture variations in tissue structure and intensity patterns, including contrast, correlation, energy, and homogeneity. To extract these texture characteristics, the Gray-Level Co-occurrence Matrix (GLCM) method was employed, which analyzes the spatial relationships between pixel intensity values. For each MRI scan, the GLCM was calculated along four orientations—0°, 45°, 90°, and 135°—and the results were averaged to generate reliable and consistent texture descriptors. The extracted features included contrast, correlation, energy, and homogeneity.
3. Shape features: Geometric properties such as area, perimeter, and eccentricity were measured from segmented brain regions, providing insights into structural characteristics.

The extracted features were concatenated to form a comprehensive feature vector, serving as input subsequent FS and classification stages. It employed MATLAB R2024a to carry out preprocessing through the following functions:

- ‘imregtform’ and ‘imwarp’ for image registration
- ‘imgaussfilt’ with sigma = 0.5 and a 3 × 3 kernel for denosing
- ‘mat2gray’ for intensities normalizing to [0, 1]
- ‘imbinarize’ for thresholding
- ‘imfill’, ‘imerode’, and ‘imdilate’ for morphological cleanup

These steps were automated using custom scripts with adjustable parameters.

### 2.4. Feature Selection Using FMO Optimizer

#### Solution Representation and Search Space in FMO Algorithm

Suppose we have a dataset with d = 10 features. In the FMO method, each solution is represented as a binary vector of length 10. For example: $X=\left[1,0,1,1,0,0,1,0,1,0\right].$ In this vector, the value ‘1’ indicates the selection of the corresponding feature and ‘0’ means its non-selection. Therefore, in the above example, features 1, 3, 4, 7, and 9 are selected. Since each feature can either be selected or not, the total search space consists of $2^{d}$ possible states. For $\left(d=10\right)$ features, this means that there are $2^{10}=1024$ potential combinations of features among which the algorithm needs to search.

The main goal of the FMO algorithm is to determine the best binary vector among these 1024 combinations; the vector that leads to the highest classification accuracy while minimizing the number of selected features. This technique helps reduce model complexity, improve computation efficiency, and prevent overfitting.

To reduce dimensionality and improve the performance of classification, the metaheuristic FMO was utilized. The process is summarized below:

Search Space Representation: Each solution was represented as a binary vector indicating the inclusion or exclusion of each feature. Algorithm 1 outlines the FMO algorithm, a bio-inspired metaheuristic designed to explore the feature selection space by simulating the adaptive hunting strategy of mantises.

In the fitness function of the FMO algorithm, a CNN-LSTM classifier was used to evaluate the model’s performance. This architecture was selected for its ability to capture spatio-temporal patterns from MRI-derived features. Based on the algorithm’s execution, 32 features were selected from the original set (such as, out of 100 extracted features, the 32 most informative ones retained). This significantly reduced the model’s dimensionality and complexity while enhancing classification accuracy. The algorithm iteratively refines a population of candidate solutions employing memory-based search, random walks, and adaptive step sizes to identify an optimal subset of features that balances classification accuracy and feature reduction.
**Algorithm 1:** Fishier Mantis Optimization (FMO) for Feature Selection.
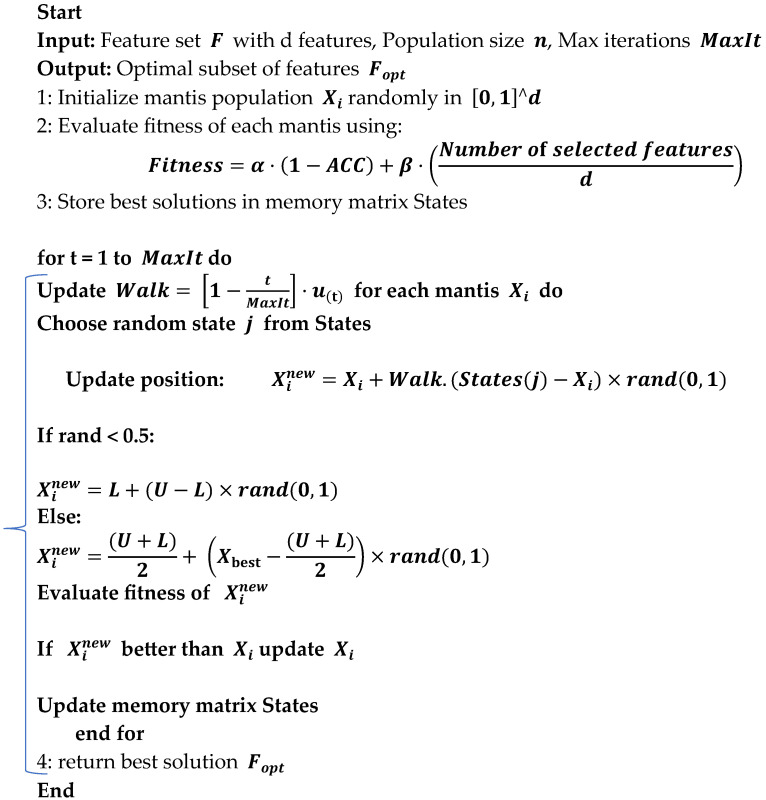


### 2.5. Fitness Function

(1) $$Fitness=\alpha \cdot \left(1-ACC\right)+\beta \cdot \left(\frac{Number\;of\;selected\;features}{d}\right)$$

where α and β are weight parameters balancing classification accuracy and feature subset size, and d is the total number of features.

Search Mechanism: The algorithm utilized random walks, memory of best solutions, and local perturbations to escape local minima and converge towards an optimum subset.

Final Output: the best-performing subset of features was selected for the final classification step.

### 2.6. Feature Selection Using FMO

In the Fishier Mantis Optimizer algorithm, the initial population is generated by positioning mantis-like agents at various random locations within the problem space. Each random position represents a potential solution. The goal is to move these initial solutions closer to the optimal solution. The initial positions are formulated according to Equation (2) and evaluated using the objective function as shown in Equation (3), and, in this context $X_{ij}$ represents the *j-th* dimension of the *i-th* solution. During the first iteration, a random population is created as described by Equation (2):

(2) $$Mantis=\left[\begin{array}{c} X_{11},X_{12},X_{13},\ldots ,X_{1d}\\ \begin{array}{c} X_{21},X_{22},X_{23},\ldots ,X_{2d}\\ \begin{array}{c} X_{31},X_{32},X_{33},\ldots ,X_{3d}\\ \vdots \end{array} \end{array}\\ X_{n1},X_{n2},X_{n3},\ldots ,X_{nd} \end{array}\right]$$

(3) $$F(Mantis)=\left[\begin{array}{c} Fittness\left(X_{11},X_{12},X_{13},\ldots ,X_{1d}\right)\\ \begin{array}{c} Fittness\left(X_{21},X_{22},X_{23},\ldots ,X_{2d}\right)\\ \begin{array}{c} Fittness\left(X_{31},X_{32},X_{33},\ldots ,X_{3d}\right)\\ \vdots \end{array} \end{array}\\ Fittness\left(X_{n1},X_{n2},X_{n3},\ldots ,X_{nd}\right) \end{array}\right]\;$$

where Mantis and F(Mantis) are matrices representing the solutions and their corresponding fitness levels. Each solution $X_{i}$ has d dimensions, such as $X_{i1},X_{i2},X_{i3},\ldots ,X_{id}$. Random solutions are generated using Equation (4):

(4) $$X_{i}=L+(U-L)\times rand(0,1)$$

In this equation, rand (0,1) generates a random vector uniformly distributed between zero and one, while. L and U denote the lower and upper bounds of the problem space, respectively.

In the Fishier Mantis Optimizer algorithm, mantises select a new position to explore and camouflage themselves. They remember several optimal states, which are organized in a matrix, denoted as *m*. This state matrix is defined as:

(5) $$States=\left[\begin{array}{c} S_{11},S_{12},S_{13},\ldots ,S_{1d}\\ \begin{array}{c} S_{21},S_{22},S_{23},\ldots ,S_{2d}\\ \begin{array}{c} S_{31},S_{32},S_{33},\ldots ,S_{3d}\\ \vdots \end{array} \end{array}\\ S_{m1},S_{m2},S_{m3},\ldots ,S_{md} \end{array}\right]$$

This matrix holds various states, with optimality assumed to be proportional to the maximum values among the solutions. The mantis retains these optimal conditions in its memory and primarily hunts in these areas.

During each update of the state matrix, mantises identify better conditions and select the optimal states from the matrix. They move towards these states using Equation (6):

(6) $$X_{i}^{new}=X_{i}+Walk\cdot (States(j)-X_{i})\times rand(0,1)$$

where $X_{i}$ is the current position, $X_{i}^{new}$ is the new position, and States(j) is a randomly chosen state. The index j is computed using Equation (7):

(7) $$j=1+\left[rand\times \left(m-1\right)\right]$$

The term Walk represents the step size, which decreases over iterations, reflecting the mantis’s approach towards the optimal solution. This step size adjustment is described by Equation (8):

(8) $$Walk=\left[1-\frac{it}{MaxIt}\right]$$

where it is the current iteration number and MaxIt is the total number of iterations. For enhanced randomness, the Chebyshev random function is used, with the step adjustment given by Equations (9) and (10):

(9) $$u_{i+1}=\cos\left(i{cos}^{-1}\left(u_{i}\right)\right),u_{1}=0.7$$

(10) $$Walk=\left[1-\frac{it}{MaxIt}\right]\cdot u_{i+1},u_{i+1}=\cos\left(i{cos}^{-1}\left(u_{i}\right)\right),u_{1}=0.7$$

### 2.7. Transfer Function

In the context of binary feature selection, the continuous position vector X is converted to binary using a transfer function (TF). The Chebyshev map helps enhance exploration by generating chaotic sequences, but a binary threshold function is employed as follows:

(11) $$\left\{\begin{array}{l} If\to X_{i}\left(j\right)\geq 0.5\to X_{i}\left(j\right)=1(Feature\;selected)\\ Else\to X_{i}\left(j\right)=0(Feature\;not\;selected) \end{array}\right.$$

This transformation allows FMO to operate in binary search space while maintaining the benefits of continuous optimization and chaotic movement control.

**Figure 3:**
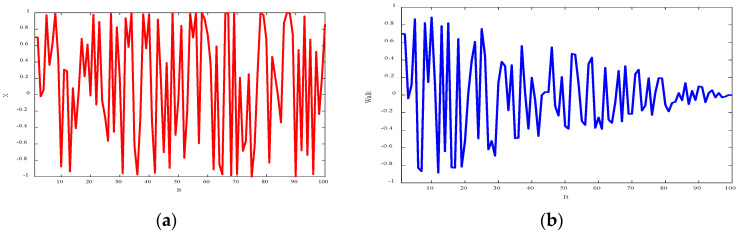
(**a**) Random sequence, and (**b**) Steps of a fishier mantis based on a random sequence.

Figure 3 shows the random sequence and step function, with the maximum number of iterations set to 100. The step size reduction transitions the search from a global to a local focus as the iterations progress.

In the optimization process, a mantis or solution may occasionally disregard previously identified optimal states in favor of exploring random positions. This approach enhances the algorithm’s global search capabilities and minimizes the risk of converging prematurely to local optima. This behavior is modeled by Equation (12). Furthermore, r, represents a random number between zero and one.

(12) $$X_{i}^{new}=\left\{\begin{array}{c} \begin{array}{cc} L+(U-L)\times rand(0,1) & r<0.5 \end{array}\\ \begin{array}{cc} \frac{L+U}{2}+(X^{*}-\frac{L+U}{2})\times rand(0,1) & 0.5\leq r \end{array} \end{array}\right.$$

Each mantis can leverage the knowledge of all previously identified optimal states. The search process involves exploring the space between the average of these optimal states and the best state achieved so far. This method is expressed in Equation (13):

(13) $$X_{i}^{new}=\left\{\begin{array}{c} \begin{array}{cc} {Walk\cdot X}_{i}+(\bar{States}-X^{*})\times rand(0,1) & rand<0.5 \end{array}\\ \begin{array}{cc} {Walk\cdot X}_{i}+(X^{*}-\bar{States})\times rand(0,1) & rand\geq 0.5 \end{array} \end{array}\right.$$

The average of the optimal states is given, in this equation, as $\bar{States}$:

(14) $$\bar{States}=\frac{\sum_{i=1}^{m}{State}_{i}}{m}$$

The number of optimal states that the algorithm needs to contend with continually drops while the mantis is edging closer to the ideal answer. Here, the decrease depends on the number of times that this process occurs (15), where *v* is the first count of those who have been busy and $m\left(t\right)$ refers to their count at the moment t.

(15) $$\;m\left(t\right)=m-\frac{m\cdot it}{MaxIt}$$

Suppose the initial feature vector consists of 6 features: $\left[F_{1},F_{2},F_{3},F_{4},F_{5},F_{6}\right]$. A solution like $X_{i}=\left[1,0,1,0,1,0\right]$ means that features f1, f3, and f5 are selected. The fitness function is as shown in the equation. For example, with Accuracy = 0.92, α = 0.7, β = 0.3, and 6 features in total, if 3 features are selected: Fitness = 0.7 × (1 − 0.92) + 0.3 × (3/6) = 0.056 + 0.15 = 0.206. The algorithm will retain this solution only if its fitness is better than the previous values.

**Figure 4:**
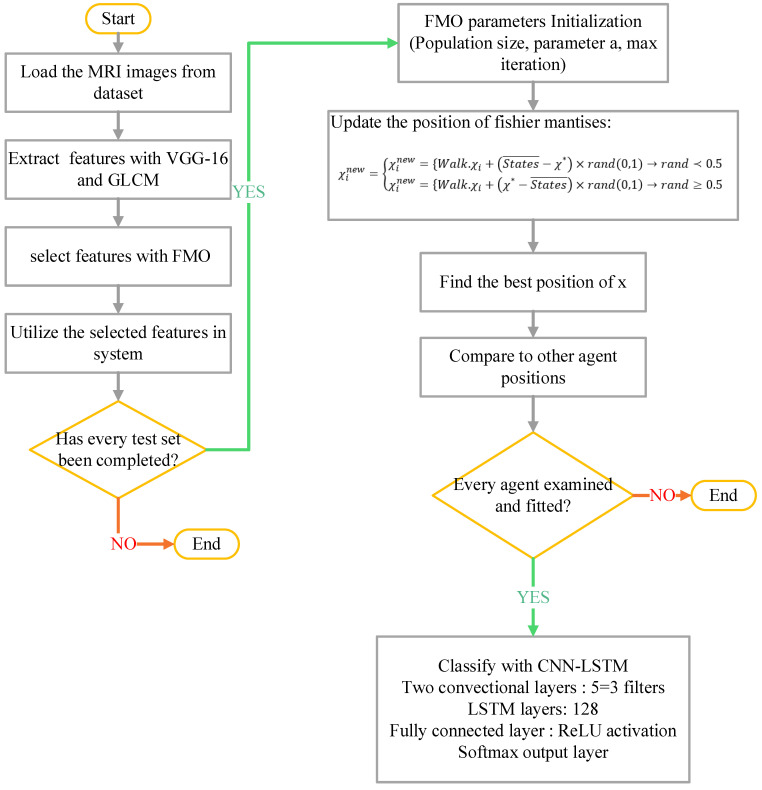
Flowchart of the proposed method.

To present the results of feature selection using the FMO algorithm, Figure 4 summarizes the proposed method for selecting features from MRI images with VGG16 and CNN models.

### 2.8. Classification with CNN-LSTM

Long Short-Term Memory (LSTM) is a type of recurrent neural network (RNN) algorithm capable of learning long-term dependencies, which is particularly useful for analysis such as medical time series and image sequences [43,44]. The classification was conducted using a hybrid Convolutional Neural Network–Long Short-Term Memory (CNN-LSTM) model with the following architecture:

- CNN Layer: Two convolutional layers with 3 × 3 filters and stride = 1, followed by max-pooling and normalization, were used to extract spatial feature.
- LSTM Layer: composed of 128 memory units for capture temporal/spatial dependencies in the data.
- Fully Connected Layer: A dense layer of 64 neurons with ReLU activation was followed by a softmax output layer for binary classification (AD vs. healthy control).

In addition to the CNN-LSTM, a basic Multi-Layer Perceptron (MLP) model was implemented for comparison. The MLP architecture comprised:

- Input layer with d neurons (equal to number of features)
- Two hidden layers, with 128 and 64 neurons respectively, both using ReLU activation
- Output layer with softmax activation for binary classification (AD vs. Healthy)
- Learning rate: 0.001
- Optimizer: Adam
- Batch size: 32, Epochs: 100

The CNN-LSTM model was optimized by the Adam optimizer with a learning rate of 0.0001. Training was performed for 100 epochs on a batch size of 32. Cross-entropy loss function was used, as well as early stopping to avoid overfitting.

### 2.9. Cross-Validation Strategy

To evaluate model performance, a 5-fold cross-validation scheme was employed:

- The data set was randomly split into five equal parts.
- In each iteration, four parts were used for training and one set part for testing.
- The final performance was obtained by averaging the results across all five folds.

### 2.10. Evaluation Indexes

Model performance was assessed using the following metrics:

- Accuracy: overall proportion of correctly classified samples.
- Sensitivity (recall): percentage of actual AD cases correctly identified.
- Specificity: percentage of healthy individuals correctly classified.
- Precision: percentage of true positives among all predicted AD cases.
- F1-score: harmonic mean of precision and recall.

To classify Alzheimer’s images, accuracy, sensitivity, and precision metrics are employed, with their respective formulas provided in Equations (16)–(18):

(16) $$Acc=\frac{TP+TN}{TP+TN+FP+FN}\times 100\%$$

(17) $$Recall=\frac{TP}{TP+FN}\times 100\%$$

(18) $$precision=\frac{TN}{TN+FP}\times 100\%$$

This study presents a fully automated and intelligent framework for Alzheimer’s disease detection using MRI images. By integrating deep and handcrafted features, optimizing the feature subset via FMO, and employing a robust CNN-LSTM classifier, the proposed system achieves high accuracy and has demonstrated strong potential for clinical application in medical image analysis. All stages are implemented in MATLAB and fully reproducible. Only authorized data from the ADNI database were used.

## 3. Results and Discussion

### 3.1. Results on ADNI Dataset

**Figure 5:**
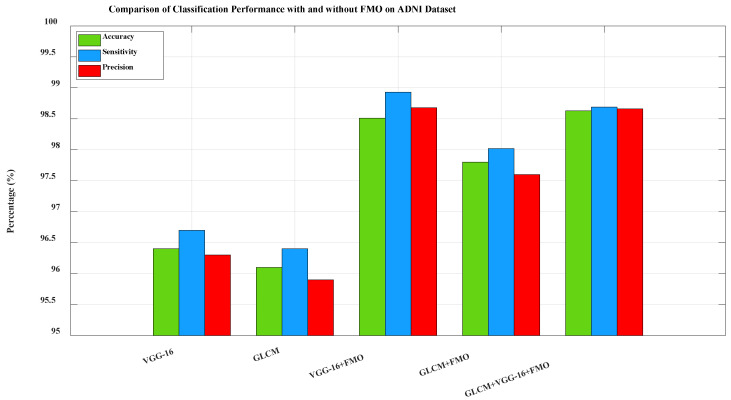
Comparison of classification performance with and without FMO on the ADNI dataset.

In all experiments presented in this section, the CNN-LSTM model was used as the final classifier to evaluate the performance of selected feature subsets. Figure 5. Comparison of classification performance on the ADNI dataset between five configurations: VGG-16 only, Gray-Level Co-occurrence Matrix (GLCM) only, VGG-16 + FMO, GLCM + FMO, and GLCM + VGG-16 + FMO. The baselines included (VGG-16 and GLCM without FMO) demonstrate the improvement in performance by the FMO algorithm. The combination of the deep and texture features optimized by the FMO significantly boosted the model’s ability to detect true AD cases while minimizing false positives. This confirms the effectiveness of the new framework when utilized for a large and heterogeneous dataset like ADNI. To further investigate the contribution of FMO in model performance, more baseline experiments were performed using only VGG-16 features and only GLCM without feature selection. These configurations, which are not part of the proposed pipeline, are shown as comparative baselines in Figure 5. The classification metrics in these configurations are significantly lower, confirming the added value of FMO in enhancing performance.

### 3.2. Results on MIRIAD Dataset

**Figure 6:**
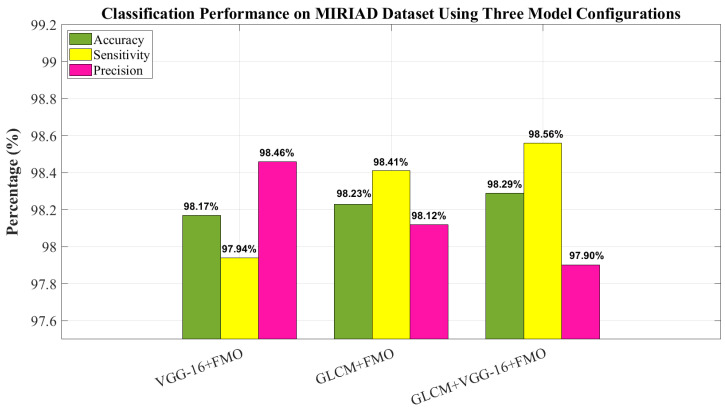
Classification performance on MIRIAD dataset using three model configurations.

Figure 6 displays the classification performance on the MIRIAD dataset using the same three configurations evaluated on the ADNI dataset. The best performance was achieved using the GLCM + VGG-16 + FMO approach, with 98.29% accuracy, 98.56% sensitivity, and 97.90% precision, as shown in Figure 6. These results further validate the model’s strong generalization capabilities, despite the smaller size and variability of the MIRIAD dataset.

### 3.3. Sensitivity Analysis of Hyperparameters

**Figure 7:**
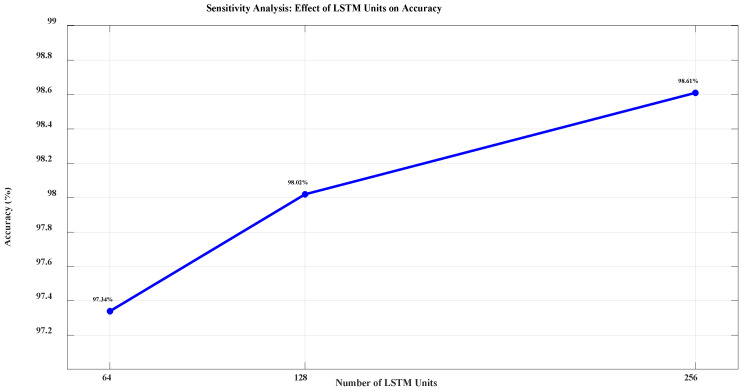
Impact of Increasing LSTM Units on Model Accuracy.

To evaluate the robustness of the proposed method, a sensitivity analysis was conducted by varying key hyperparameters of the FMO algorithm (population size, iterations) and CNN-LSTM architecture (number of LSTM memory units, filter size in CNN). The model’s accuracy remained within ±1.2% of the baseline performance, confirming high stability. Figure 7 illustrates the effect of varying the LSTM units (64, 128, 256) on classification accuracy.

Figure 7 clearly shows that an increase in the number of memory units in the LSTM layer leads to a significant improvement in classification accuracy. The highest accuracy (98.61%) is achieved using 256 LSTM units. This sensitivity analysis emphasizes the critical importance of optimizing the number of LSTM units to obtain better model performance.

Table 3 is compiled to compare the performance of three classification architectures used on the ADNI dataset (MLP: Multi-Layer Perceptron, LSTM: Long Short-Term Memory, CNN: Convolutional Neural Network, FMO: Fisher Mantis Optimization). The comparison is based on standard evaluation metrics like Accuracy, Precision, Sensitivity/Recall, and F1 Score, all expressed as percentages.

The MLP-LSTM model, trained and tested using the same cross-validation protocol as the other models, serves as a baseline. The role of this model is to evaluate the incremental impact of more sophisticated convolutional architectures and feature optimization techniques on improving classification performance.

### 3.4. Comparison with Existing Deep Learning Models

**Figure 8:**
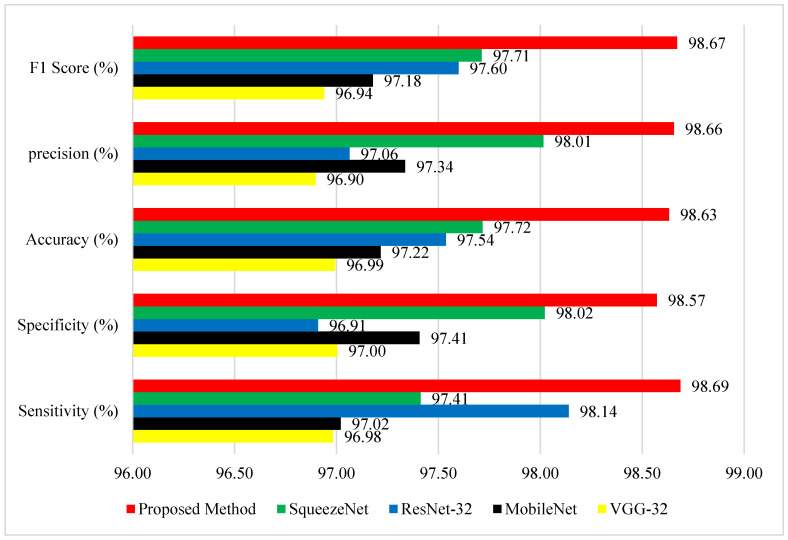
Performance comparison of the new model (GLCM + VGG16 + FMO) versus four DL models on the ADNI dataset, evaluated on Accuracy, Precision, Sensitivity, Specificity, and F1-Score.

The GLCM + VGG16 + FMO model demonstrated outstanding performance on the ADNI dataset, as evidenced by the results in Figure 8. The accuracy of the proposed method greatly exceeds that of the methods presented in studies [39,42] for Alzheimer’s disease detection.

Figure 8 provides a detailed comparison of performance among the proposed method and four well-known deep learning models—SqueezeNet, ResNet-32, MobileNet, and VGG-32—on five evaluation measures: F1 Score, Precision, Accuracy, Specificity, and Sensitivity. The proposed method (red) performs better than all other methods in all metrics, reflecting its strength and improved classification capability. Most importantly, it achieves the highest F1 Score of 98.67%, signifying excellent trade-off between precision and recall. With respect to precision, the suggested approach achieves 98.66%, which is far ahead of the competitors, with SqueezeNet being the closest at 98.01%, and MobileNet and VGG-32 at 97.34% and 96.90%, respectively. Concerning the measurement of general accuracy, the suggested approach achieves 98.63%, once again outperforming ResNet-32 (97.54%) and SqueezeNet (97.72%). Most significantly, the new method attains a specificity of 98.57%, demonstrating its excellent ability to correctly identify negative cases, which is extremely important in the minimization of false positives. This impressive advance over ResNet-32 (96.91%) and MobileNet (97.41%) can significantly enhance the model’s efficiency. Finally, the proposed method attains a sensitivity (true positive rate) of 98.69%, surpassing even the solid performance of ResNet-32 (98.14%) and SqueezeNet (97.41%). These results clearly show that the proposed method not only guarantees well-balanced performance on all main evaluation metrics but also attains considerable gains against widely applied structures. The gains are particularly apparent in precision and specificity, thus rendering this approach highly effective in applications requiring high detection quality together with low misclassifications, such as in medical diagnosis, anomaly detection, or industrial inspection systems. The visual evidence shows that the approach proposed in this paper is a stable and balanced solution to challenging classification problems. Reference [40] includes CNN-based feature extraction combined with classifiers such as SVM and Decision Trees. This method outperforms the others in terms of all major parameters, particularly sensitivity and precision. Table 4 shows performance comparison of deep learning models with and without Fisher Mantis Optimization (FMO) for Alzheimer’s in diagnosis on ADNI dataset (DL: Deep Learning, FMO: Fisher Mantis Optimization, AUC: Area Under the Curve, CNN: Convolutional Neural Network). All metrics are reported as percentages.

The performance metrics presented in Table 4 reflect a comprehensive analysis of various deep learning models for AD diagnosis, specifically focusing on VGG-16, SqueezeNet, MobileNet, and ResNet50, both with and without the integration of the Fisher Mantis Optimization (FMO) algorithm. Among all configurations, the VGG16-FMO model demonstrates the most outstanding diagnostic performance, achieving the highest sensitivity (98.93%), specificity (98.64%), accuracy (98.51%), precision (98.68%), F1-score (98.53%), and AUC (91.03%). These results highlight the approach’s exceptional ability to correctly identify both Alzheimer’s and non-Alzheimer’s cases with minimal error. Close behind is the SqueezeNet-FMO model, which also performs remarkably well, particularly in terms of sensitivity (98.79%) and precision (98.59%), showcasing the effectiveness of FMO even in lightweight architectures. MobilNet-FMO and ResNet50-FMO also show significant performance improvements over their original versions, with MobilNet-FMO achieving an accuracy of 98.26% and an F1-score of 98.28%, while ResNet50-FMO maintains solid values across all metrics, albeit slightly lower. In contrast, the baseline models without FMO consistently report lower results. For example, the standard VGG16 model has a sensitivity of 97.89%, accuracy of 97.47%, and AUC of 77.60%, indicating a noticeable performance gap compared to its FMO-enhanced counterpart. The differences are even more pronounced in SqueezeNet, MobileNet, and ResNet50, whose AUC scores without FMO fall below 74%, signifying weaker discriminatory capabilities. Overall, the table clearly demonstrates that the integration of the Fisher Mantis Optimization algorithm significantly boosts the diagnostic performance of all tested models, with VGG16-FMO standing out as the most effective framework for AD detection.

### 3.5. ROC Curve Analysis

**Figure 9:**
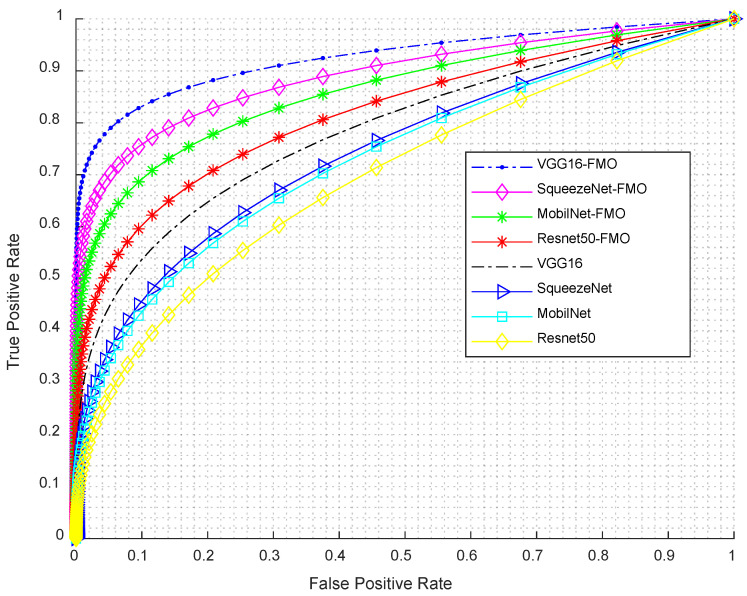
ROC curves of VGG16, SqueezeNet, MobileNet, and ResNet50 with and without FMO, showing improved performance with FMO integration.

Figure 9 presents the ROC curves comparing the performance of VGG16, SqueezeNet, MobileNet, and ResNet50 models, both with and without FMO. Models integrated with FMO consistently demonstrate improved true positive rates across all false-positive rate thresholds.

The ROC curves in Figure 9 illustrate the classification performance of various deep learning models—VGG16, SqueezeNet, MobileNet, and ResNet50—with and without the integration of the Fishier Mantis Optimization (FMO) algorithm. The true positive rate (sensitivity) is plotted against the false-positive rate for each model configuration, providing a comprehensive view of their diagnostic capabilities. From the graph, it is evident that models enhanced with the FMO algorithm consistently outperform their baseline counterparts, as their curves are closer to the top-left corner, indicating a higher rate of true positive detections with fewer false positives. Notably, the VGG16-FMO model exhibits the best overall performance, with the steepest curve and the highest true positive rate across all false-positive rate thresholds, demonstrating the strong effectiveness of FMO in improving detection accuracy. SqueezeNet-FMO also performs significantly better than the standard SqueezeNet, highlighting FMO’s impact even on lightweight architectures. MobileNet-FMO and ResNet50-FMO similarly show noticeable improvements over their original forms, although to a slightly lesser degree than VGG16-FMO. In contrast, the original versions of these models, particularly MobileNet and ResNet50, show relatively lower performance, with curves that fall below their optimized counterparts, reflecting their comparatively limited sensitivity and precision without the aid of FMO. Overall, this ROC analysis clearly demonstrates the beneficial influence of the Fishier Mantis Optimization algorithm across multiple architectures, particularly when paired with deep convolutional networks like VGG16.

### 3.6. Comparative Literature Review

Recent studies using ADNI and MIRIAD datasets report accuracies ranging from 93 to 97% using methods such as PCA + SVM [31], CNN + SVM [45], and 3D-CNN + BiLSTM [46]. The new framework surpasses these methods with 98.63% accuracy (Figure 8). Table 5 compares the proposed method with related recent studies on the ADNI dataset, highlighting differences in methodology, accuracy, and the use of feature selection techniques. (CNN: Convolutional Neural Network, SVM: Support Vector Machine, BiLSTM: Bidirectional Long Short-Term Memory, FMO: Fisher Mantis Optimization).

### 3.7. Comparison of Metaheuristic Algorithms

Table 6 shows the performance comparison of different metaheuristic algorithms—Particle Swarm Optimization (PSO) [47], Ant Colony Optimization (ACO [48]), Grey Wolf Optimizer (GWO) [49], and Bitterling Fish Optimization (BFO) [50]—integrated with Long Short-Term Memory (LSTM) and the Visual Geometry Group-16 model (VGG-16) in terms of Sensitivity, Specificity, Accuracy, Precision, and F1 Score.

The performance comparison of various metaheuristic optimization algorithms combined with LSTM and VGG-16 for classification tasks reveals that all models deliver exceptionally high results across key performance metrics, indicating their effectiveness and robustness. Among the combinations, the Bitterling Fish Optimization (BFO) algorithm integrated with LSTM and VGG-16 achieved the highest overall performance, with a sensitivity of 98.07%, specificity of 98.72%, accuracy of 98.39%, precision of 98.78%, and F1-score of 98.43%. This suggests that BFO is particularly efficient in accurately detecting true positives while maintaining a low false-positive rate, contributing to its superior F1-score. Close behind, the Gray Wolf Optimization (GWO) approach recorded slightly lower but still impressive metrics, with a sensitivity of 98.01%, specificity of 98.66%, accuracy of 98.34%, precision of 98.63%, and F1-score of 98.32%, highlighting its strong balance between sensitivity and specificity. The Ant Colony Optimization (ACO) method also demonstrated robust results, with a sensitivity of 98.41% and an F1-score of 98.27%, showing its competitive performance. Meanwhile, the Particle Swarm Optimization (PSO)-based model delivered a sensitivity of 97.94%, specificity of 98.41%, and an F1-score of 98.20%, which, although slightly lower than the others, still underscores its viability for high-accuracy tasks. Overall, these findings emphasize that the integration of advanced optimization algorithms with LSTM and VGG-16 significantly enhances classification performance, with BFO emerging as the most effective among the evaluated methods.

## 4. Conclusions

In this study, we proposed an effective approach for identifying AD by combining the power of deep learning and optimization methods. The VGG-16 model was utilized for feature extraction, enabling the efficient capture of intricate patterns within MRI images, which are crucial for early Alzheimer’s detection. To address the challenge of high-dimensional data, we incorporated the Fisher Mantis optimization algorithm (FMO), which provided an efficient means for feature dimension reduction, thereby enhancing the classification performance while minimizing computational complexity. Our results demonstrate the effectiveness of the proposed model in terms of accuracy and robustness, outshining traditional methods by providing more reliable and efficient predictions. The combination of VGG-16 and FMO not only improved the performance of the diagnosis system but also highlighted the potential of optimizing deep learning models using biologically-inspired algorithms. This approach paves the way for the development of advanced diagnostic systems for AD, with potential applications in clinical settings for timely intervention and improved disease management. Future work will focus on further refining the feature extraction and optimization phases, exploring additional datasets, and assessing the model’s real-time application in clinical environments to enhance its generalizability and practicality.

## Figures and Tables

**Table 1:** Comparison of State-of-the-Art Models for Alzheimer’s Diagnosis.

Ref.	Feature Selection Method	Classifier	Dataset	Accuracy (%)	Feature Rate (%)	Notes
[30]	GA	Fisher SVM (Linear & RBF)	ADNI (136 subjects)	96.32	98.52	Competitive with state-of-the-art; data fusion boosts performance
[31]	GA	SVM	ADNI (458 subjects)	93.01	96.8	Strong performance, MCI conversion prediction, 10-fold CV
[32]	Manual Feature Extraction (MR & PET only)	Linear SVM	ADNI (202 subjects)	93.2	93.3	Combines MRI, FDG-PET, CSF; kernel-based fusion outperforms concatenation
[33]	Sparse Group Lasso, ICA	Sparse Group Lasso (SGL)	AD: 77, HC: 173 (Total: 250)	0.98	0.95	FA clustered via ICA is best; strong performance from tractography and TBSS measures
[34]	Pixel/Voxel Analysis (via ICA & graph features)	Sparse Group Lasso	77 AD, 173 HC	92	N/A	Best result from FA ICA clustering; used multiple diffusion-based methods and graph features
[35]	Linguistic Feature Extraction (taxonomy-guided)	SVM (Linear, RBF, Poly)	Alzheimer’s patients and healthy controls (exact size not specified)	~91.0	N/A	Proposed a new taxonomy of linguistic features; SVM (linear) showed best classification performance
[36]	Aggregative multi-filter gene selection (degree, bottleneck, RF, DISR) + ranking aggregation	Logistic Regression	Regression GSE48350, GSE36980, GSE132903, GSE118553, GSE5281; Validation: GSE109887	86.8	50 genes (from 803 overlapping DEGs)	Multi-filter gene ranking integrated with feature selection, robust across multiple brain regions; validated externally; pathway analysis confirms biological relevance
[37]	Transfer Learning (TL) with radiomic + TL features; Fine-tuning 2D CNNs (ResNet18/50/101) on 3D MRI	6 classifiers in General Approach; fine-tuned CNNs in Deep Approach	80 3T MRI scans (with historical 1.5T scans for scenario A)	Scenario A: 99%; Scenario B: 83%	N/A	Addresses MRI domain shift; TL boosts AD diagnosis; fine-tuned 2D models adapted to 3D MRI data

**Table 2:** Characteristics of the ADNI and MIRIAD datasets with training/test split (70/30).

Dataset	Class	Total Samples	Training Samples (70%)	Test Samples (30%)
**ADNI**	**Alzheimer’s (AD)**	**314**	**220**	**94**
**ADNI**	**Control (NC)**	**427**	**299**	**128**
**MIRIAD**	**Alzheimer’s (AD)**	**46**	**32**	**14**

**Table 3:** Comparative performance of MLP-LSTM, CNN-LSTM, and VGG16-based models (ADNI dataset).

Model	Accuracy (%)	Precision (%)	Sensitivity (%)	F1 Score (%)
MLP-LSTM	97.84	97.88	97.9	97.89
CNN-LSTM	98.23	98.41	98.29	98.35
VGG16 + FMO	98.51	98.68	98.93	98.53

**Table 4:** Performance comparison of DL models for Alzheimer’s diagnosis with and without FMO on the ADNI dataset.

Feature Extractor + Feature Selector + Classifier	Sensitivity (%)	Specificity (%)	Accuracy (%)	Precision (%)	F1-Score (%)	AUC (%)
**VGG16 + FMO + softmax**	**98.93**	**98.64**	**98.51**	**98.68**	**98.53**	**91.03**
**SqueezeNet + FMO + softmax**	**98.79**	**98.55**	**98.3**	**98.59**	**98.31**	**87.83**
**MobilNet + FMO + softmax**	**98.47**	**97.96**	**98.26**	**97.94**	**98.28**	**84.85**
**Resnet50 + FMO + softmax**	**98.19**	**97.73**	**97.69**	**97.72**	**97.67**	**80.73**
**Without Feature Selection (FMO)**						
**VGG16 + softmax**	**97.89**	**97.67**	**97.47**	**97.69**	**97.51**	**77.6**
**SqueezeNet + softmax**	**97.72**	**96.75**	**97.37**	**96.83**	**97.36**	**73.6**
**MobilNet + softmax**	**97.01**	**96.62**	**97.04**	**96.58**	**97.03**	**72.53**
**Resnet50 + softmax**	**96.14**	**96.21**	**96.96**	**96.19**	**96.95**	**69.03**

**Table 5:** Comparison with Related Works.

Ref.	Method	Dataset	Accuracy (%)	Feature Selection
[45]	CNN + SVM	ADNI	96.34	No
[46]	3D-CNN + BiLSTM	ADNI	97.45	Yes
This study	GLCM + VGG16 + FMO	ADNI	98.63	Yes (FMO)

**Table 6:** Performance of PSO, ACO, GWO, and BFO with LSTM and VGG-16 across key metrics.

Method	Sensitivity (%)	Specificity (%)	Accuracy (%)	Precision (%)
VGG-16 + FOM + PSO + LSTM	97.94	98.41	98.17	98.46
VGG-16 + FOM + ACO + LSTM	98.41	98.04	98.23	98.12
VGG-16 + FOM + GWO + LSTM	98.01	98.66	98.34	98.63
VGG-16 + FOM + BFO + LSTM	98.07	98.72	98.39	98.78

## Data Availability

The datasets generated and analyzed during the current study are available from the corresponding author on reasonable request.
